# Serotonin transporter-deficient mice display enhanced adipose tissue inflammation after chronic high-fat diet feeding

**DOI:** 10.3389/fimmu.2023.1184010

**Published:** 2023-07-13

**Authors:** Johannes Hoch, Niklas Burkhard, Shanshan Zhang, Marina Rieder, Timoteo Marchini, Vincent Geest, Krystin Krauel, Timm Zahn, Nicolas Schommer, Muataz Ali Hamad, Carolina Bauer, Nadine Gauchel, Daniela Stallmann, Claus Normann, Dennis Wolf, Rüdiger Eberhard Scharf, Daniel Duerschmied, Nancy Schanze

**Affiliations:** ^1^ Cardiology and Angiology, Medical Center – University of Freiburg, Faculty of Medicine, University of Freiburg, Freiburg, Germany; ^2^ Department of Cardiology, Angiology, Haemostaseology and Medical Intensive Care, University Medical Centre Mannheim, Medical Faculty Mannheim, Heidelberg University, Mannheim, Germany; ^3^ Translational Cardiology, Department of Cardiology, Inselspital, Bern, Switzerland; ^4^ Department of Psychiatry and Psychotherapy, Medical Center - University of Freiburg, Faculty of Medicine, University of Freiburg, Center for Basics in Neuromodulation, Faculty of Medicine, University of Freiburg, Freiburg, Germany; ^5^ Program in Cellular and Molecular Medicine, Boston Children's Hospital, Harvard Medical School, Boston, MA, United States; ^6^ Division of Experimental and Clinical Hemostasis, Hemotherapy, and Transfusion Medicine, Blood and Hemophilia Comprehensive Care Center, Institute of Transplantation Diagnostics and Cell Therapy, Heinrich Heine University Medical Center, Düsseldorf, Germany; ^7^ European Center for AngioScience (ECAS) and German Center for Cardiovascular Research (DZHK) Partner Site Heidelberg/Mannheim, Mannheim, Germany

**Keywords:** serotonin, metabolic syndrome, obesity, adipose tissue, inflammation, serotonin transporter

## Abstract

**Introduction:**

Serotonin is involved in leukocyte recruitment during inflammation. Deficiency of the serotonin transporter (SERT) is associated with metabolic changes in humans and mice. A possible link and interaction between the inflammatory effects of serotonin and metabolic derangements in SERT-deficient mice has not been investigated so far.

**Methods:**

SERT-deficient (*Sert* ^-/-^) and wild type (WT) mice were fed a high-fat diet, starting at 8 weeks of age. Metabolic phenotyping (metabolic caging, glucose and insulin tolerance testing, body and organ weight measurements, qPCR, histology) and assessment of adipose tissue inflammation (flow cytometry, histology, qPCR) were carried out at the end of the 19-week high-fat diet feeding period. In parallel, *Sert* ^-/-^ and WT mice received a control diet and were analyzed either at the time point equivalent to high-fat diet feeding or as early as 8-11 weeks of age for baseline characterization.

**Results:**

After 19 weeks of high-fat diet, *Sert* ^-/-^ and WT mice displayed similar whole-body and fat pad weights despite increased relative weight gain due to lower starting body weight in *Sert* ^-/-^. In obese *Sert* ^-/-^ animals insulin resistance and liver steatosis were enhanced as compared to WT animals. Leukocyte accumulation and mRNA expression of cytokine signaling mediators were increased in epididymal adipose tissue of obese *Sert* ^-/-^ mice. These effects were associated with higher adipose tissue mRNA expression of the chemokine monocyte chemoattractant protein 1 and presence of monocytosis in blood with an increased proportion of pro-inflammatory Ly6C+ monocytes. By contrast, *Sert* ^-/-^ mice fed a control diet did not display adipose tissue inflammation.

**Discussion:**

Our observations suggest that SERT deficiency in mice is associated with inflammatory processes that manifest as increased adipose tissue inflammation upon chronic high-fat diet feeding due to enhanced leukocyte recruitment.

## 1 Introduction

Obesity can lead to the development of non-alcoholic fatty liver disease (NAFLD), insulin resistance, type-2 diabetes mellitus, and cardiovascular diseases (1, 2). Obesity-related sequelae are promoted by a chronic low-grade inflammation, which is characterized by a persistent release of proinflammatory mediators and accumulation of leukocytes (e.g., macrophages and T cells), especially in visceral adipose tissue (1–3).

The biogenic amine serotonin (5-hydroxytryptamine) exerts multiple functions in the central nervous system but also plays a significant role in peripheral tissues. Peripherally acting serotonin has been shown to aggravate diet-induced obesity, insulin resistance and NAFLD in mice (4). Several lines of evidence suggest that serotonin also contributes to leukocyte recruitment and function during inflammatory processes (5–9).

**Figure 1:**
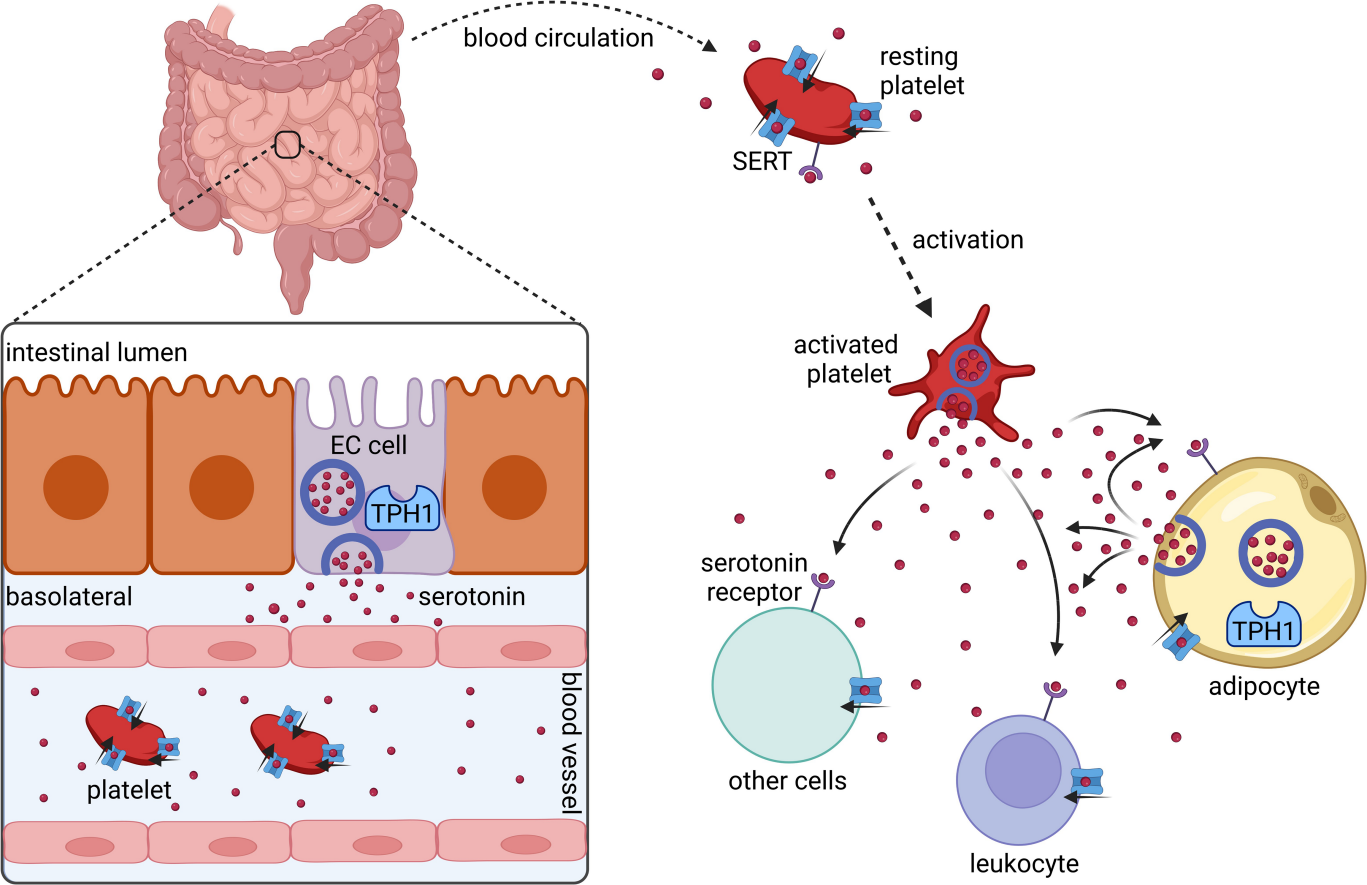
Synthesis and distribution of peripherally acting serotonin. Serotonin is synthesized and released by intestinal enterochromaffin cells (EC cells) that express tryptophan hydroxylase 1 (TPH1). In the blood stream serotonin is taken up by platelets *via* the serotonin transporter (SERT). Platelets release serotonin upon their activation. Several cell types including platelets, leukocytes, and adipocytes express serotonin receptors and SERT. Adipocytes synthesize and secrete serotonin locally in adipose tissues. Created with BioRender.com.

The vast majority of serotonin is produced by enterochromaffin cells in the gut (10) (**Figure 1**). Upon release into the blood stream, serotonin is taken up by circulating platelets that serve as a major serotonin carrier (11). Upon platelet activation, serotonin is secreted (11). However, more recent data show that serotonin synthesis also occurs in adipocytes (12, 13).

Interestingly, knockout studies of the key enzyme in peripheral serotonin synthesis (tryptophan hydroxylase I) suggest that the aforementioned metabolic and inflammatory effects of serotonin in obese mouse models can be mediated almost entirely by adipocyte-derived serotonin, independent of gut-derived serotonin (4, 9, 13, 14).

Actions of serotonin are mainly mediated by cell membrane receptors, which are for example expressed on cells of the innate and adaptive immune system but also on adipocytes (5, 15). In this regard, the bioavailability of serotonin is largely influenced by the serotonin transporter SERT that facilitates serotonin uptake or reuptake into many different cell types, including adipocytes, platelets, and leukocytes (5, 12). Serotonin (re-)uptake *via* SERT reduces the amount of extracellular serotonin that can activate cell membrane receptors and subjects serotonin to enzymatic degradation in several cell types (5, 12, 15, 16).

In mice, a lack of SERT expression can lead to enhanced expansion of white adipose tissue, decreased glucose tolerance, and hepatic steatosis (17–20). Genetic and epigenetic alterations in the activity or abundance of the SERT in humans are associated with increased body mass index and type-2 diabetes (21–23). Likewise, pharmacological inhibition of SERT activity by antidepressants such as selective serotonin reuptake inhibitors (SSRI) is reported to be associated with an increased risk of type-2 diabetes (24). With respect to the often-observed coincidence of obesity and depression in humans and the frequent use of SSRI in this population, interrelations of altered SERT function and metabolic and/or cardiovascular consequences of obesity are of major interest (25–27).

Whether metabolic derangements in SERT-deficiency are driven by adipose tissue inflammation has not yet been investigated in detail. We hypothesized, that SERT knockout with resulting dysregulation of extracellular serotonin levels can aggravate adipose tissue inflammation thereby promoting obesity-related insulin resistance and hepatic steatosis in the setting of diet-induced obesity.

In this study, we fed SERT-deficient mice a high-fat diet (HFD) for 19 weeks and analyzed visceral adipose tissue for inflammatory responses in relation to body weight and energy metabolism. We demonstrate that SERT deficient mice show elevated visceral adipose tissue inflammation, insulin resistance and liver steatosis after chronic HFD feeding. Our observations suggest that SERT deficiency is associated with inflammatory processes due to an increase in leukocyte recruitment into adipose tissue upon diet-induced obesity.

## 2 Methods

### 2.1 Animals

Male *Sert* ^−/−^ mice on a C57BL/6J background and age-matched wild-type C57BL/6J *Sert* ^+/+^ (WT) mice were housed in the local animal facility at a 12 h light/dark cycle with *ad libitum* access to water and food. Mice were fed either a high-fat diet (HFD, D12451, 45 kJ% fat, Sniff Spezialdiäten) starting at 8 weeks of age or a control diet (CD, 3437 PXL15M/R, 4,5 kJ% fat, Granovit AG) for 19 weeks (HFD: WT n = 6-7, Sert^-/-^ n = 6-9; CD: n = 3-4 per genotype). Body weight was determined weekly. At the end of the study, mice were sacrificed upon anesthesia with ketamine (100 mg/kg body weight) and xylazine (20 mg/kg body weight) followed by terminal cardiac blood collection. In parallel, a group of *Sert* ^-/-^ and WT mice receiving a control diet was analyzed as early as 8-11 weeks of age for baseline characterization (n=5 per genotype). All experiments were conducted in accord with the German animal protection law. All procedures had been approved by the federal authorities in Baden-Wurttemberg, Germany (File # 35-9185.81/G-19/132).

### 2.2 Insulin and glucose tolerance testing

Mice were fasted for 12 or 2 hours prior to glucose or insulin tolerance testing, respectively. After initial blood collection *via* tail vein puncture, 1 g glucose per kg body weight or 0.5 U human insulin per kg body weight were injected intraperitoneally. Subsequent blood collections were performed after 15, 30, 45, 60, 90, and 120 minutes, and glucose concentrations were measured using a blood glucose meter (ACCU-CHECK Aviva, Roche Diabetes Care GmbH). Results are indicated as area of the curve with baseline subtraction of t = 0 minutes to account for differences in fasting glucose levels (28).

### 2.3 Metabolic caging

Food intake, VCO_2_ production, VO_2_ consumption, respiratory exchange ratio (RER), heat dissipation and locomotor activity were assessed using metabolic cages (Comprehensive Lab Animal Monitoring System, Columbus Instruments) for 48 hours (24 hours adaptation time, 24 hours measurements).

### 2.4 Sample preparation and flow cytometric analyses

Epididymal fat was collected, immediately weighed, and transferred to cold Dulbecco’s phosphate-buffered saline with Ca^2+^ and Mg^2+^ (DPBS, Gibco, ThermoFisher Scientific) supplemented with 0.5% bovine serum albumin (BSA). The tissue was minced, digested with 3 mg collagenase I dissolved in 2 ml DPBS containing 0.5% BSA for 45 minutes at 37°C and subsequently transferred through a 100 µm cell strainer.

Spleens were passed through a 70 µm cell strainer into 0.1% BSA in DPBS.

After cutting off the epiphyses, the bone marrow was rinsed from the femora with 0.1% BSA in DPBS and passed through a 70 µm cell strainer.

After centrifugation (spleen, bone marrow: 5 minutes, 350 x *g*; epididymal white adipose tissue: 3 minutes, 1000 x *g*, room temperature (RT)), red blood cell lysis was performed with red blood cell (RBC) lysis buffer (BioLegend) for 5 minutes on ice and stopped by adding 12.5 ml of DPBS. Cells were washed, centrifuged, resuspended in 0.1% BSA in DPBS, and stored on ice. Leukocyte concentrations were determined in a Neubauer chamber and were adjusted to 5.5 million (fat, bone marrow) or 11 million (spleen) leukocytes per ml. After blocking unspecific Fc Receptor-mediated antibody binding (TruStain FcX™ anti-mouse CD16/32, Biolegend), 90 µl of the samples were incubated with viability dye (Viability Dye eFluor™ 506, ThermoFisher Scientific) and flow cytometry antibodies (anti-mouse CD45 PacBlue; anti-mouse CD11b PerCP; anti-mouse F4/80 PE-Cy7; anti-mouse CD115 APC; anti-mouse Ly6C PE; anti-mouse Ly6G APC-Cy7; anti-mouse CD3 FITC or PE; anti-mouse CD19 FITC or APC-Cy7; anti-mouse NK1.1 FITC or APC; anti-mouse CD4 PerCP; anti-mouse CD8 FITC; anti-mouse CD41 PECy7; all from BioLegend) for 25 minutes on ice in the dark. After another washing step, cells were fixed (Cellfix, BD Biosciences) and stored in the dark at 4°C until flow cytometric analysis.

EDTA blood (5 µl) was incubated with 95 µl of RBC lysis buffer (5 minutes, RT), and leukocytes were counted in a Neubauer chamber. For flow cytometric analysis, 40 µl of EDTA blood were diluted in 200 µl DPBS with 0.1% BSA. After blockade of Fc receptors, specific antibody staining was performed in analogy to the procedure used for spleen, bone marrow, and fat. Staining was followed by red blood cell lysis and fixation (Lyse/fix buffer, BD Biosciences) for 30 minutes at RT in the dark. The samples were then centrifuged (8 minutes, RT, 500 x *g*) and cell pellets were resuspended in 300 µl DPBS. Samples were stored at 4°C in the dark until flow cytometric analysis. Flow cytometry data were acquired on a BD LSRFortessa (Becton, Dickinson and Company).

Representative gating strategies are shown in **Supplementary Figure S1**.

### 2.5 Analysis of mRNA expression

RNA extraction was performed with the Aurum Total RNA Mini Kit (Bio-Rad) according to the manufacturer’s instructions. Purity and concentration of the RNA-preparations were assessed by NanoDrop (ThermoFisher Scientific). Subsequent cDNA synthesis was carried out with the iScript™ cDNA Synthesis Kit (Bio-Rad) following the manufacturer’s instructions. mRNA expression was analyzed using iQ™ SYBR® Green Supermix, and gene specific primers (**Supplemental Table S1**). Gene expression was calculated using the 2^-ΔΔCt^ method.

### 2.6 Serotonin concentration in serum and plasma

To assess “free” and “total” (platelet-stored) serotonin in the circulation, its concentrations were determined in plasma and serum, respectively. Blood was allowed to stay at RT for at least 3 h to generate serum. After centrifugation (5 minutes, 1300 x *g*), the supernatant was collected and stored at -80°C. To obtain plasma, the collected blood was mixed with EDTA (Invitrogen). Samples of EDTA blood (400 µl) were carefully mixed with 1 µg/ml prostacyclin (PGI_2_) and centrifuged (5 minutes, RT, 600 x *g*). The collected supernatant was again centrifuged (5 minutes, RT, 1000 x *g*), and the remaining supernatant was stored at -80°C. Plasma and serum serotonin were measured using the Serotonin Research ELISA (LDN Labor Diagnostika Nord) or the Serotonin ELISA Fast Track (LDN Labor Diagnostika Nord), respectively. The manufacturer’s instructions were followed.

### 2.7 Histology

Immediately after dissection, epididymal fat and livers were fixed in phosphate-buffered 4% formaldehyde solution (Carl Roth) for 24 hours. Subsequently, fixed tissues were embedded in paraffin, sectioned, and mounted onto microscope slides. Hematoxylin and eosin (HE) staining was performed according to standard protocols in the Core Facility for Histopathology and Digital Pathology at the Medical Center - University of Freiburg.

### 2.8 Statistics and data presentation

Statistical analyses were performed with GraphPad Prism Version 9. Normal distribution was tested using Shapiro-Wilk test. In case of normal distribution, data are presented as mean ± standard error of the mean (SEM), otherwise median with interquartile range (IQR) is indicated. Statistical outliers were detected by ROUT test. For comparison of two groups, an unpaired two-tailed student’s t-test (for normally distributed data) or Mann-Whitney test (nonparametric) was performed. Welch’s correction was applied in case of unequal variances between the groups.

## 3 Results

### 3.1 SERT-deficiency in young mice is associated with monocytosis and increased fat mass, but not increased adipose tissue inflammation

**Figure 2:**
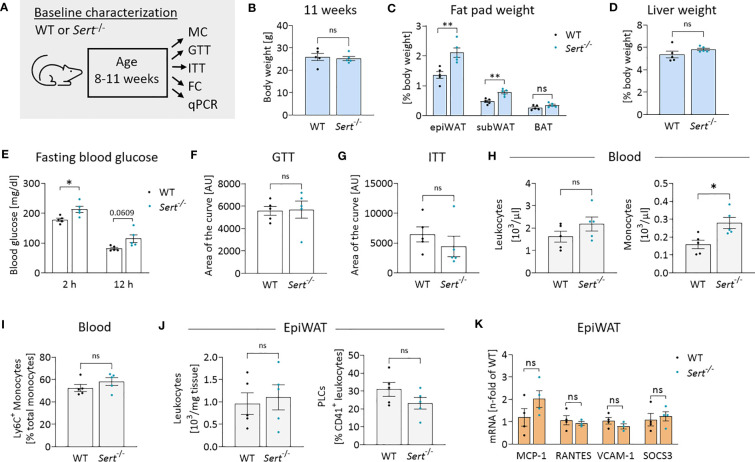
Energy metabolism and inflammation in SERT-deficient mice at baseline. **(A)** Experimental set up for baseline characterization of male mice at an age of 8-11 weeks fed a control diet (CD). WT, wild type; *Sert* ^-/-^, serotonin transporter knockout; MC, metabolic caging; GTT, glucose tolerance testing; ITT, insulin tolerance testing; FC, flow cytometry; qPCR, quantitative polymerase chain reaction. **(B)** Body weight of 11 weeks old mice. **(C)** Fat pad weights in relation to whole body weight in 11 weeks old mice; epiWAT, epididymal white adipose tissue; subWAT, subcutaneous white adipose tissue; BAT, brown adipose tissue. **(D)** Liver weight in relation to whole body weight of 11 weeks old mice. **(E)** Fasting blood glucose after 2 or 12 hours of food deprivation in CD-fed mice. **(F)** Glucose tolerance testing (GTT) at 10 weeks of age. Displayed is the area of the curve from GTT data obtained after 12 hours of fasting. **(G)** Insulin tolerance testing (ITT) at 11 weeks of age. Indicated is the area of the curve from insulin tolerance testing after 2 hours of fasting. **(H)** Circulating leukocyte and monocyte (Viab^-^, CD45^+^, CD3^-^, CD19^-^, NK1.1^-^, CD11b^+^, Ly6G^-^, F4/80^-^) counts detected by FC at 11 weeks of age. **(I)** Proportion of Ly6C^+^ monocytes depicted as percentage of the entire monocyte population in blood, determined by FC. **(J)** Leukocytes and platelet-leukocyte complexes (PLC; Viab^-^, CD45^+^, CD41^+^) detected by FC in the stromal vascular fraction from epiWAT of CD-fed mice at 11 weeks of age. **(K)** qPCR on whole tissue epiWAT mRNA-preparations from 11 weeks old WT (black dots) and *Sert* ^-/-^ (blue dots) mice feeding on a CD; MCP-1, monocyte chemoattractant protein 1; RANTES, regulated and normal T cell expressed and secreted; VCAM-1, vascular cell adhesion molecule 1, SOCS3; suppressor of cytokine signaling 3. **(B–J)** n=5 per group. **(K)** n=4 per group. Results are shown as mean ± SEM. Asterisks indicate statistical significance: * p<0.05; **p<0.01; ns, not statistically significant.

In the baseline study, *Sert* ^-/-^ mice displayed increased fat pad weights despite similar whole-body weights in comparison to the WT animals at 11 weeks of age (**Figures 2A–C**). Liver weights were not significantly different between both genotypes (**Figure 2D**). While fasting blood glucose was increased in *Sert* ^-/-^ mice at baseline (**Figure 2E**), there was no difference in glucose tolerance or insulin sensitivity (**Figures 2F, G**). At baseline, *Sert* ^-/-^ tended to show less physical activity than WT mice, while respiratory exchange ratio, heat dissipation and food intake were similar between the groups (**Supplemental Figures S2A-D**). Moreover, circulating leukocytes were not significantly different between both genotypes. A monocytosis in *Sert^-^* ^/-^ mice was observed at baseline (**Figure 2H**). The percentages of circulating pro-inflammatory Ly6C+ monocytes did not differ significantly between genotypes (**Figure 2I**). Interestingly, monocytosis was associated with a decreased number of splenic but not bone marrow monocytes (**Supplemental Figures S2F, G**). Leukocyte numbers and platelet-leukocyte complexes in epiWAT (**Figure 2J**, **Supplemental Figure S2E**) were similar in both groups. The expression of the chemokines MCP-1 (monocyte chemoattractant protein 1) and RANTES (regulated and normal T cell expressed and secreted) as well as endothelial adhesion molecule VCAM-1 (vascular cell adhesion molecule 1), and cytokine signaling mediator SOCS3 (suppressor of cytokine signaling 3) was not altered between the genotypes (**Figure 2K**).

### 3.2 No accumulation of leukocytes in epididymal adipose tissue of SERT-deficient mice after 19-weeks of control diet feeding

**Figure 3:**
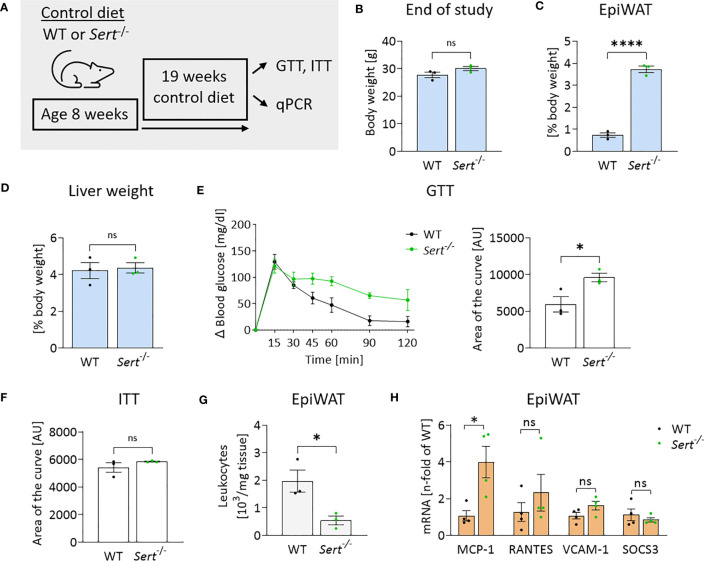
Energy metabolism and inflammation in SERT-deficient mice after 19 weeks of control diet feeding. **(A)** Experimental set up of the control diet study. Male 8-week-old wild type (WT) or serotonin transporter knockout (*Sert* ^-/-^) mice received a control diet (CD) for 19 weeks, data displayed in B-H are from mice at the end of the 19 weeks CD feeding period. GTT, glucose tolerance testing; ITT, insulin tolerance testing; qPCR, quantitative polymerase chain reaction. **(B)** Body weight of *Sert* ^-/-^ or WT mice. **(C)** Weight of epididymal white adipose tissue (epiWAT) in relation to whole body weight. **(D)** Liver weight in relation to whole-body weight. **(E)** Glucose tolerance testing (GTT), displayed are the glucose excursion curves (left) and the data determined as area of the curve (right). **(F)** Depicted is the area of the curve from insulin tolerance testing (ITT). **(G)** Leukocyte numbers (10^3^/mg adipose tissue) in the stromal vascular fraction of epiWAT. **(H)** qPCR on whole tissue epiWAT mRNA-preparations from WT (black dots) or *Sert* ^-/-^ (green dots) mice; MCP-1, monocyte chemoattractant protein 1; RANTES, regulated and normal T-cell expressed and secreted; VCAM-1, vascular cell adhesion molecule 1; SOCS3, suppressor of cytokine signaling 3. **(B–G)** n=3 per group, **(H)** n=4 per group. Results are shown as mean ± SEM. Asterisks indicate statistical significance: * p<0.05; **** p<0.0001; ns, not statistically significant.

After 19 weeks of control diet (CD)-feeding, *Sert* ^-/-^ had accumulated more epididymal white adipose tissue (epiWAT) than WT mice (**Figure 3C**), while whole-body weights and liver weights were similar between genotypes (**Figures 3B, D**). Decreased glucose tolerance (but not insulin sensitivity) was observed in the CD-fed *Sert* ^-/-^ at the end of the feeding interval (**Figures 3E, F**). No leukocyte accumulation in epiWAT of CD-fed *Sert* ^-/-^ was observed (**Figure 3G**). MCP-1 expression was higher in the epiWAT of *Sert* ^-/-^ than in WT mice, while there was no increase in RANTES, SOCS3 or VCAM-1 expression (**Figure 3H**).

### 3.3 Disturbance of glucose homeostasis and extent of liver steatosis are more pronounced in obese SERT-deficient mice

**Figure 4:**
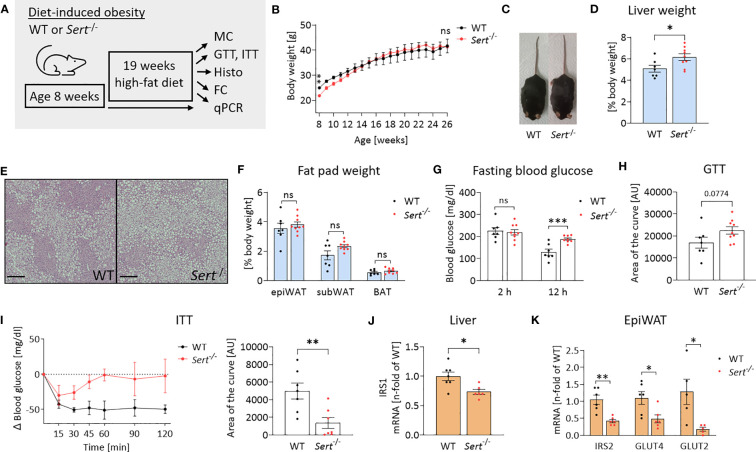
Body weight and energy metabolism in obese SERT-deficient mice. **(A)** Experimental set up of the high-fat diet (HFD) study. WT, wild type; *Sert* ^-/-^, serotonin transporter knockout; MC, metabolic caging; GTT, glucose tolerance testing; ITT, insulin tolerance testing; Histo, histology; hematoxylin and eosin staining (HE), FC, flow cytometry; qPCR, quantitative polymerase chain reaction. **(B)** Body weight increase of male *Sert* ^-/-^ or WT mice feeding on a HFD. **(C)** Representative pictures of *Sert* ^-/-^ and WT at 19 weeks of HFD. **(D–K)** data from mice after chronic HFD feeding. **(D)** Liver weight in relation to body weight. **(E)** Representative images of liver sections stained with HE. Scale bar = 200 µm. **(F)** Weight of epididymal white adipose tissue (epiWAT), subcutaneous white adipose tissue (subWAT), and brown adipose tissue (BAT) in relation to body weight. **(G)** Fasting blood glucose after 2 hours or 12 hours of food deprivation. **(H)** Glucose tolerance testing (GTT). Indicated is the area of the curve of the GTT carried out after a 12 h fasting period. **(I)** Insulin tolerance testing (ITT). Depicted are the glucose excursion curves (left) and the calculated area of the curve (right) of the ITT carried out after 2 h of fasting. **(J)** Hepatic mRNA expression as detected by qPCR; IRS1: insulin receptor substrate 1. **(K)** qPCR on whole tissue epiWAT mRNA-preparations from WT (black dots) and *Sert* ^-/-^ (red dots) mice; IRS2, insulin receptor substrate 2; GLUT4/GLUT2, glucose transporter 4/2. **(B, D, F, G, H)** WT n=7, *Sert* ^-/-^ n=9; **(I)** n=7 per group; **(J)** WT n=7, *Sert* ^-/-^ n=6; **(K)** n=6 per group. Results are shown as mean ± SEM. Asterisks indicate statistical significance: * p<0.05; **p<0.01; *** p<0.001; ns, not statistically significant.

At the beginning of high-fat diet (HFD) feeding (8 weeks of age), *Sert* ^-/-^ mice displayed lower body weights than the age-matched WT controls (**Figures 4A, B**). After accelerated body weight gain during the first 4 weeks on HFD, the weight curves had a less steep progression. This resulted in similar body weights in both groups at the end of the study (**Figures 4B, C**). However, obese *Sert* ^-/-^ mice had increased relative liver weights and increased hepatic lipid accumulation compared to the WT (**Figures 4D, E**), whereas relative fat pad weights were similar in both groups at the end of the study (**Figure 4F**). Obese SERT-deficient mice displayed lower glucose tolerance and insulin sensitivity, as compared to the WT (**Figures 4G–I**). This was accompanied by a stronger decrease (i.e., shift toward lipid utilization) in the respiratory exchange ratio (RER) in obese *Sert* ^-/-^ (**Supplemental Figure S2H**). The mRNA expression of insulin receptor substrate (IRS) 1 and 2 were decreased in obese *Sert* ^-/-^ livers and epiWAT, respectively (**Figures 4J, K**). Moreover, GLUT2- and GLUT4-mRNA levels were decreased in obese *Sert* ^-/-^ epiWAT (**Figure 4K**).

### 3.4 Enhanced leukocyte accumulation in visceral adipose tissue of obese SERT-deficient mice

**Figure 5:**
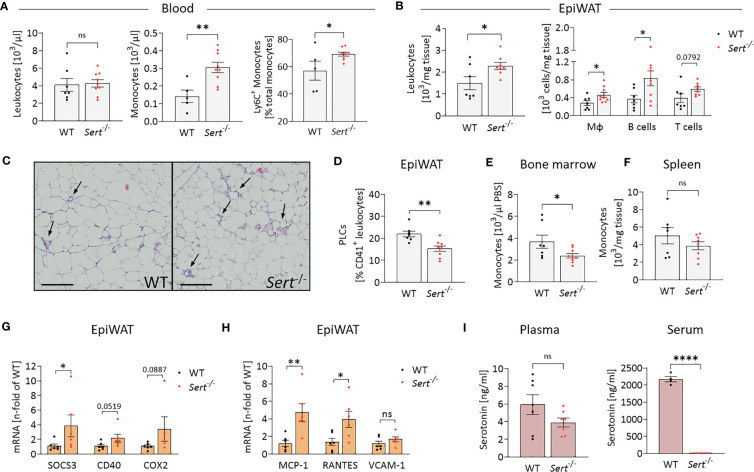
Increased leukocyte infiltration into epididymal white adipose tissue of obese SERT-deficient mice. Eight-week-old male *Sert* ^-/-^ and WT mice consumed a HFD for 19 weeks. **(A-I)** data from mice after chronic HFD feeding. **(A)** Circulating leukocyte and monocyte counts (10^3^/µl) and proportion of Ly6C^+^ monocytes depicted as percentage of the entire monocyte population, determined by flow cytometry (FC). Monocytes were gated as Viab^-^, CD45^+^, CD3^-^, CD19^-^, NK1.1^-^, CD11b^+^, Ly6G^-^, F4/80^-^. **(B)** Leukocyte, macrophage (Viab^-^, CD45^+^, CD3^-^, CD19^-^, NK1.1^-^, CD11b^+^, Ly6G^-^, F4/80^+^), B-cell (Viab^-^, CD45^+^, CD3^-^, CD19^+^) and T-cell (Viab^-^, CD45^+^, CD19^-,^CD3^+^) numbers (10^3^/mg tissue) analyzed by FC in the stromal vascular fraction (SVF) of epididymal white adipose tissue (epiWAT). **(C)** Representative images of epiWAT stained with hematoxylin and eosin, arrows indicate crown-like structures, scale bar = 200 µm. **(D)** Platelet-leukocyte complexes (PLC; Viab^-^, CD45^+^, CD41^+^) in the epiWAT SVF as determined by FC. **(E)** Bone marrow monocytes (Viab^-^, CD45^+^, CD3^-^, CD19^-^, NK1.1^-^, CD11b^+^, Ly6G^-^, F4/80^-^) per µl PBS (used to flush femur samples) measured by FC. **(F)** FC Analysis of monocytes (Viab^-^, CD45^+^, CD3^-^, CD19^-^, NK1.1^-^, CD11b^+^, Ly6G^-^, F4/80^-^) per mg of spleen tissue. G/H: qPCR on whole tissue epiWAT mRNA-preparations from WT (black dots) and *Sert* ^-/-^ (red dots) mice; SOCS3, suppressor of cytokine signaling 3; CD40, cluster of differentiation 40; COX2, cyclooxygenase 2; MCP-1, monocyte chemoattractant protein 1; RANTES, regulated and normal T cell expressed and secreted; VCAM-1, vascular cell adhesion molecule 1. **(I)** Plasma and serum serotonin concentrations as measured by ELISA. A, B, D, E, F: WT n=7, *Sert* ^-/-^ n=9; **(G, H)** n=6 per group; **(I)** plasma n=7 per group, serum WT n=4, *Sert* ^-/-^ n=5. Results are shown as mean ± SEM. Asterisks indicate statistical significance: * p<0.05; **p<0.01; **** p<0.0001; ns, not statistically significant.

After HFD-feeding, *Sert* ^-/-^ and WT animals had similar total circulating leukocyte counts, whereas the percentage of monocytes was increased in the blood of *Sert* ^-/-^ (**Figure 5A**). Additionally, the proportion of pro-inflammatory Ly6C^+^ monocytes was higher in the circulation of *Sert* ^-/-^ (**Figure 5A**). Total epiWAT leukocyte counts, macrophages, B cells and T cells were increased in *Sert* ^-/-^ mice (**Figure 5B**). In line with this, HE-staining of epiWAT sections revealed an abundance of crown-like structures, indicative of macrophages engulfing adipocytes, in *Sert^-/-^* (**Figure 5C**). The percentage of platelet-leukocyte complexes in the epiWAT was lower in *Sert* ^-/-^ than in WT mice (**Figure 5D**). Bone marrow monocyte counts were decreased in obese *Sert^-/-^* compared to WT (**Figure 5E**), whereas monocyte numbers in the spleen were not significantly different between both genotypes (**Figure 5F**). An increased expression of genes related to cytokine signaling was detected in epiWAT of obese *Sert* ^-/-^ (**Figure 5G**). In addition, the chemokines MCP-1 and RANTES were upregulated (**Figure 5H**). The expression of the endothelial adhesion molecule VCAM-1 was similar in both genotypes (**Figure 5H**).

### 3.5 Serum serotonin is decreased in SERT-deficient mice

Plasma serotonin concentrations did not differ significantly between *Sert* ^-/-^ and WT mice. By contrast, serum serotonin was decreased below detection limit of the assay in *Sert* ^-/-^ mice, verifying the lack of SERT-mediated serotonin uptake and storage in platelets (**Figure 5I**).

## 4 Discussion

Here, we demonstrate that deficient SERT expression in chronic HFD fed animals leads to impaired systemic glucose homeostasis and increased hepatic steatosis compared to WT animals. This difference occurs despite similar body and fat depot weights of both genotypes at the end of the HFD study (**Figures 4B, F**). SERT-deficient animals have increased fat mass at the end of the control diet study (**Figures 3B, C**), which is consistent with previous reports (18–20). By contrast, no such an effect is observed after feeding the HFD for 19 weeks (**Figure 4F**). A possible explanation for the obtained differences is provided by identification of increased adipose tissue dysfunction in obese SERT-deficient mice. This contention is based on two other observations made in this study. First, adipose dysfunction becomes evident from enhanced inflammation, displaying increased numbers of macrophages, B cells and T cells (**Figure 5B**), and abundance of crown-like structures (**Figure 5C**). Second, insulin sensitivity of the adipose tissue in SERT deficiency is decreased compared to WT mice. This is shown by lower expression of the insulin-sensitive glucose transporter GLUT4 and lower expression of the insulin receptor substrate 2 (IRS2) which is an important component of insulin-dependent intracellular signal transduction (**Figure 4K**) (29, 30). As a result, the adipose tissue growth of SERT-deficient mice under HFD is restricted. This deteriorated adipose tissue function contributes to impaired hepatic insulin sensitivity (as demonstrated by decreased IRS1 expression, **Figure 4J**) and pronounced hepatic steatosis (as evident by abundant lipid droplets and higher liver weight) (**Figures 4D, E**). An inverse relationship between epididymal adipose tissue mass and liver lipid accumulation in murine obesity has been described previously (31). Inflammation and dysfunction of adipose tissue in the context of ongoing caloric excess lead to impaired adipose tissue lipid handling with consequently increased circulating free fatty acids and release of adipokines (32, 33). This so-called metaflammation can significantly contribute to the development of hepatic insulin resistance and steatosis (32, 34, 35). The reduced insulin-dependent inhibition of hepatic glucose production in turn intensifies hyperglycemia in obese *Sert* ^-/-^ mice (**Figure 4G**) (36). Together, decreased insulin sensitivity of liver and adipose tissue contributes to an overall impaired glucose homeostasis and, in particular, to increased insulin resistance in obese SERT-deficient mice (**Figure 4I**).

We also show in this study that increased adipose tissue inflammation of SERT-deficient animals after consumption of the HFD is due to increased leukocyte recruitment into adipose tissue. The increased number of macrophages in *Sert* ^-/-^ adipose tissue (**Figure 5B**) is caused by a combination of present monocytosis, characterized by an increased proportion of proinflammatory Ly6C^+^ monocytes (**Figure 5A**) together with an increased adipose tissue expression of the chemokine MCP-1 (**Figure 5H**). MCP-1 is produced, for example, by adipocytes (37). In an MCP-1-dependent manner, Ly6C^+^ monocytes migrate into visceral adipose tissue and differentiate into adipose tissue macrophages, which are involved in removal of dying fat cells and debris from the adipose tissue, e.g., in crown-like structures (38).

The increased number of T cells in adipose tissue of SERT-deficient animals is caused by increased expression of the chemokine RANTES (**Figure 5H**). RANTES which can also be produced by adipocytes is known to promote T cell accumulation in adipose tissue (39–41). T cells may also act as a trigger for macrophage infiltration into the adipose tissue in SERT deficiency in our study. It has been shown that CD8^+^ T cells precede macrophage infiltration and promote macrophage accumulation in obese adipose tissue (42). In addition, consistent with data from others (43), our study demonstrates that platelets and/or platelet-leukocyte complexes can be detected in adipose tissue (**Figure 5D**). This observation suggests that platelets may also be sources of the chemokines MCP-1 and RANTES, thus contributing to leukocyte recruitment (44–46). This platelet effect on leukocyte recruitment is independent of serotonin, as demonstrated here by the *Sert* ^-/-^ phenotype (**Figure 5I**).

Our findings also suggest that the leukocyte populations (macrophages, B cells and T cells, **Figure 5B**) having infiltrated the obese adipose tissue contribute to adipose tissue insulin resistance in SERT-deficient mice by releasing proinflammatory mediators. These proinflammatory mediators can activate pathways that include the cytokine signaling mediator SOCS3 and the pro-inflammatory receptor CD40 (as shown in **Figure 5G**). The mediators thereby interfere with intracellular insulin signaling as demonstrated by decreased IRS2 and GLUT4 expression in epididymal fat tissue (**Figure 4K**). SOCS3 and CD40-related pathways have been widely described to inhibit insulin receptor-activated pathways in adipocytes (47–49). The association between inflammation and insulin resistance is supported by data from the control diet feeding arm of our study (**Figure 3**). Here, despite greater fat mass (**Figure 3C**), the mice do not develop adipose tissue inflammation (**Figures 3G, H**) and, correspondingly, display no insulin resistance (**Figure 3F**). The difference in glucose tolerance between the genotypes consuming the control diet (**Figure 3E**) might be caused, for example, by altered glucose effectiveness (glucose-stimulated glucose uptake independent of insulin) in SERT-deficient mice compared to WT (28). One reason for this finding may be the lower percentage of lean body mass in SERT deficiency. A similar effect has also been observed previously (19).

Another observation of this study is that *Sert* ^-/-^ mice display an increased inflammatory state. Even at young age, there are cellular biomarkers of pre-existing inflammation in SERT-deficient mice, as evident by blood monocytosis (**Figure 2H**). However, enhanced adipose tissue inflammation only becomes overt upon HFD-consumption (**Figure 5B, G**) while it is absent in mice that had received a control diet (**Figure 2J, K**; **Figure 3G, H**; **Supplementary Figure S2E**). Therefore, SERT deficiency appears to make the mice more susceptible to external inflammatory triggers such as HFD feeding. In support of this observation is a study demonstrating that expression of inflammatory cytokines can be upregulated in the myocardium of *Sert* ^-/-^ mice after experimental myocardial infarction. By contrast, there were no differences observed in non-operated mice (50). Furthermore, it has been shown in a mouse model of chemically induced colitis that SERT deficiency can increase infiltration of inflammatory cells into the mucosa and submucosa (51). A possible reason why adipose tissue inflammation only becomes overt upon HFD feeding, as in our study, is that local adipose tissue serotonin synthesis and concentrations are increased in diet-induced obese mice (13). Adipocyte-derived serotonin exerts effects directly in visceral adipose tissue thereby affecting energy metabolism, enhancing macrophage infiltration, and inflammatory gene expression during the development of diet-induced obesity (9, 13, 14). These effects can be mediated for example *via* the serotonin receptors 5-hydroxytryptamine receptor 2A and 2B (9, 14). The missing SERT-mediated termination of serotonin signaling may even potentiate these effects in *Sert* ^-/-^ mice.

There are several limitations of our study that should be considered when interpreting the data. First, we did not establish a time course of HFD feeding intervals. Therefore, we cannot assess inflammatory *vs*. metabolic effects in a chronological sequence and interdependency. Our data obtained from the combination of different feeding protocols suggest that there is an increased susceptibility to inflammation in SERT-deficient mice already at a young age under steady state conditions. However, we cannot rule out that insulinemia in *Sert* ^-/-^ mice reinforces or contributes to adipose tissue inflammation itself (19, 52). Previously suggested mechanisms by which lack of SERT function influences glucose homeostasis include lowered estrogen levels in SERT-deficient mice and a gut microbiome profile that resembles a pattern known for obesity (20, 53, 54). Furthermore, future analyses of mediators in *Sert* ^-/-^ mice are needed to explore the origin and corresponding contribution of different adipose tissue-associated cell types (such as adipocytes, endothelial cells, leukocytes, or platelets) to increased chemokine expression. This is particularly interesting because many different cell types are affected in the global SERT knockout. For example, SERT is expressed by endothelial cells, adipocytes, platelets and leukocytes (5, 12, 15). Furthermore, different types of serotonin receptors are found on these cell types (5, 9, 12, 15, 55)

Taken together, our study provides insights into metabolic changes related to serotonin under conditions of a deficient serotonin transporter (SERT). Using a knockout mouse model, we demonstrate that SERT deficiency aggravates obesity-related adipose tissue inflammation. The inflammatory response of adipose tissue is shown to result from increased leukocyte recruitment into obese visceral adipose tissue, thereby exacerbating adipose tissue dysfunction, disturbing systemic glucose homeostasis, and causing liver steatosis. In perspective, these findings may have broader implications with regard to individuals with SERT polymorphisms and also for the use of selective serotonin reuptake inhibitor (SSRI) class anti-depressants, specifically in obese patients.

## Data availability statement

The raw data supporting the conclusions of this article will be made available by the authors, without undue reservation.

## Ethics statement

The animal study was reviewed and approved by Regierungspräsidium Freiburg Referat 35 - Veterinärwesen, Lebensmittelüberwachung Bertoldstraße 43 79098 Freiburg i. Br.

## Author contributions

Conceptualization: NaS, MR, DD, DW. Study design: NaS, MR, DD, DW. Experiments: JH, NB, SZ, TM, VG, KK, TZ, NiS, MH, CB, NG, CN, DS, TZ, NaS. Data analysis: JH, NB, SZ, MR, VG, NaS. Statistics: JH, NB, MR, NaS. Writing - original draft preparation: NS. Writing – review, revision, and editing: NaS, RS. All authors contributed to the article and approved the submitted version.
