# Supplementary material for: Serotonin transporter-deficient mice display enhanced adipose tissue inflammation after chronic high-fat diet feeding
Source: Front Immunol. 2023 Jul 13;14:1184010. doi: 10.3389/fimmu.2023.1184010 (PMC10372416; doi:10.3389/fimmu.2023.1184010)
Supplement: Supplementary file 1 [file DataSheet_1.docx]

Supplementary Material

Serotonin transporter-deficient mice display enhanced adipose tissue inflammation after chronic high-fat diet feeding

**Johannes Hoch^1†^, Niklas Burkhard^1†^, Shanshan Zhang^1,2^, Marina Rieder^1,3^, Timoteo Marchini^1^, Vincent Geest^1^, Krystin Krauel^1,2^, Timm Zahn^1^, Nicolas Schommer^1^, Muataz Ali Hamad^1^, Nadine Gauchel^1^, Daniela Stallmann^1^, Claus Normann^4^, Dennis Wolf^1^, Rüdiger Eberhard Scharf^2,5,6^, Daniel Duerschmied^1,2,7^, Nancy Schanze^1,2*^**

*** Correspondence:** Nancy Schanze: Nancy.schanze@umm.de

# Supplementary Figures


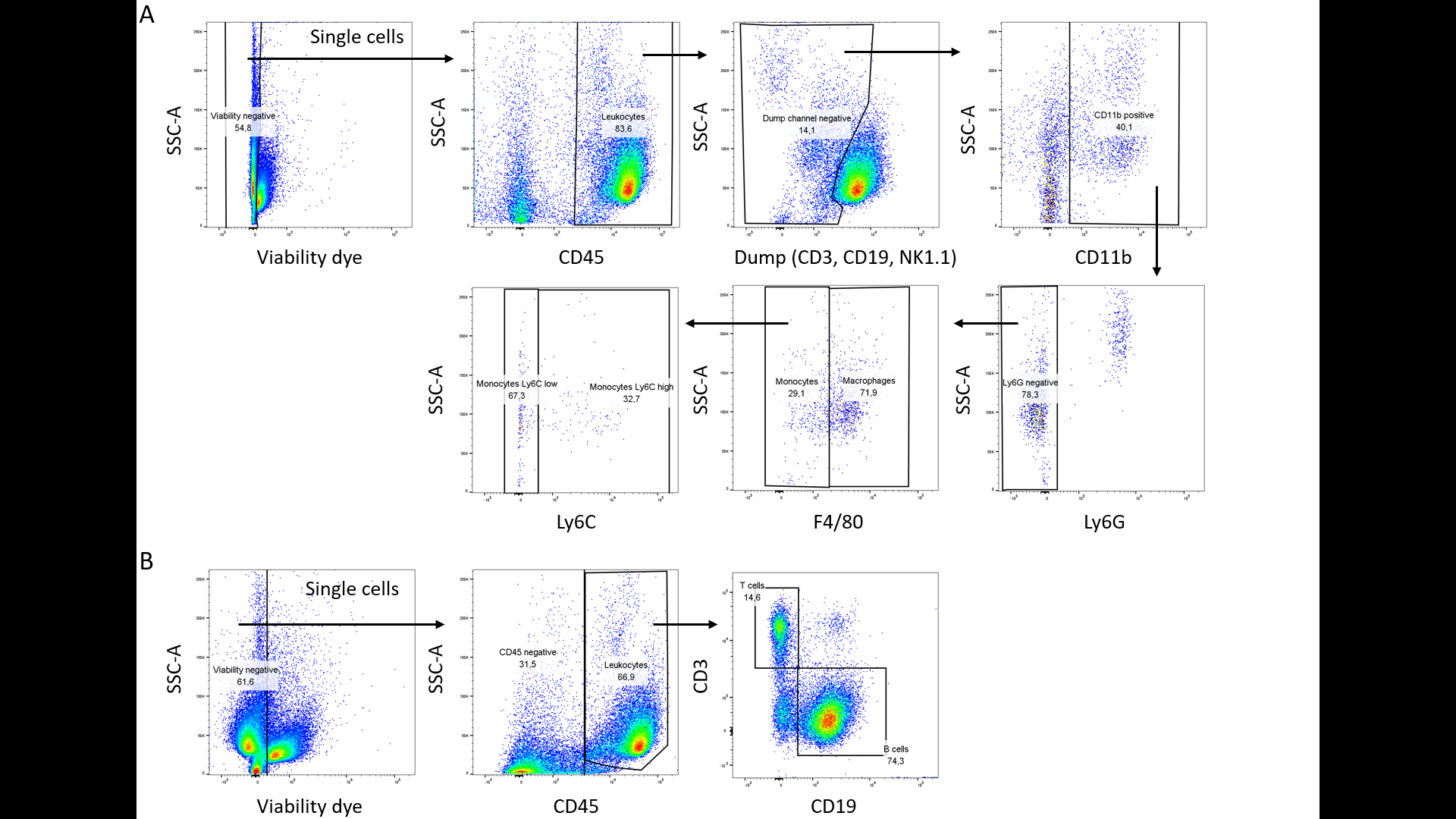


**Supplementary Figure S1: Representative gating strategies for the analysis of flow cytometry data.** A: Flow cytometry gating strategy for myeloid cells. B: Flow cytometry gating strategy for lymphocytes.

**
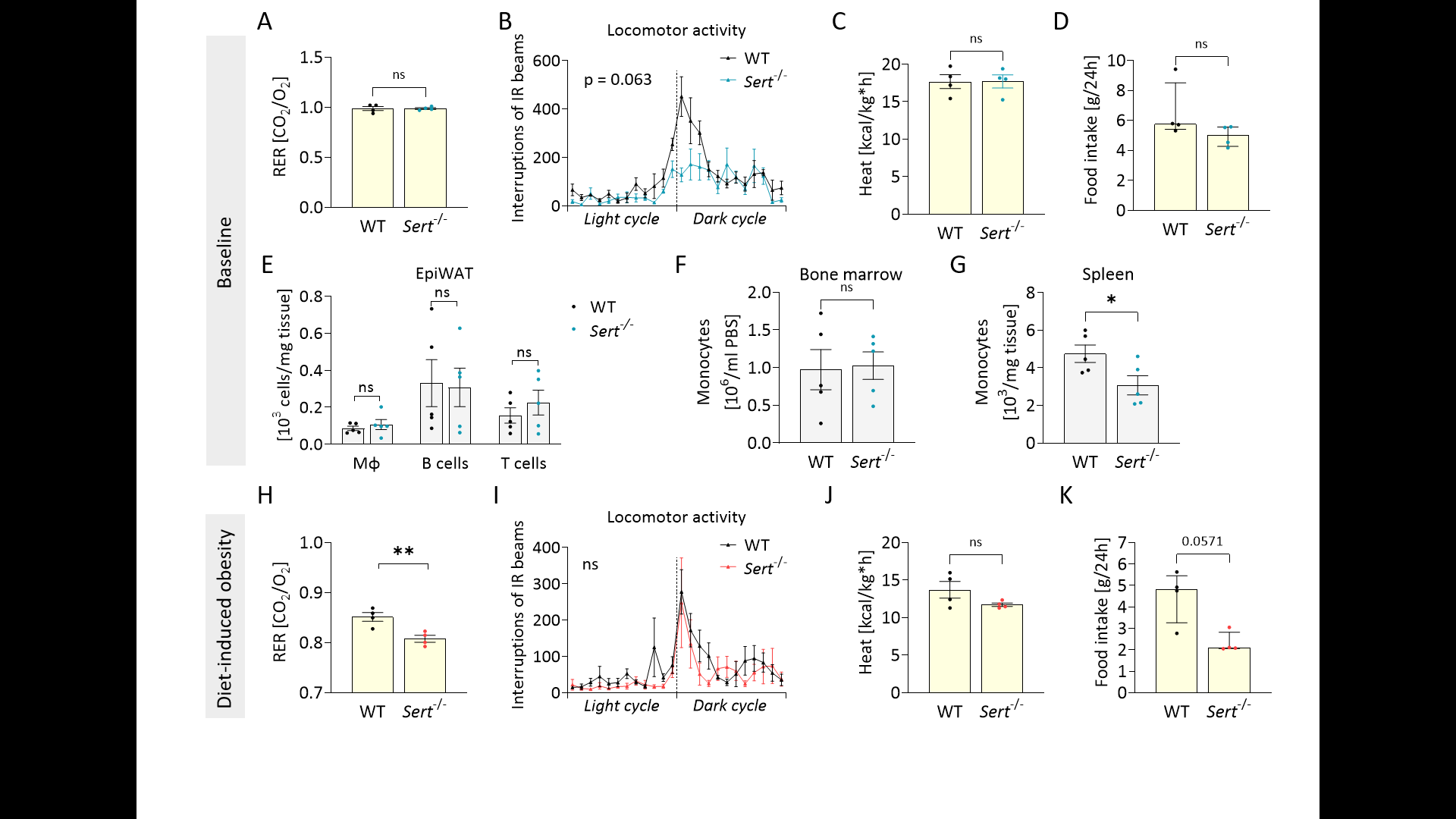
**

**Supplementary Figure S2: Additional characteristics of SERT-deficient mice at baseline and after HFD-feeding . A-D:** Metabolic caging data from mice at baseline (8/9 weeks of age) receiving a control diet. . A: Respiratory exchange ratio (RER). B: Locomotor activity measured as interruption of infrared (IR) beams. C: Heat dissipation per hour. D: Food intake per 24 hours. E-G: Flow cytometric data from mice at baseline (11 weeks of age). E: Macrophages (Viab^-^, CD45^+^, CD3^-^, CD19^-^, NK1.1^-^, CD11b^+^, Ly6G^-^, F4/80^+^), B cells (Viab^-^, CD45^+^, CD3^-^, CD19^+^) and T cells (Viab^-^, CD45^+^, CD19^-,^ CD3^+^) in the stromal vascular fraction of epididymal white adipose tissue (epiWAT) from wild type (WT,black dots) and *Sert*^-/-^ mice (blue dots), depicted as 10^3^ cells per mg of adipose tissue. F: Monocyte (Viab^-^, CD45^+^, CD3^-^, CD19^-^, NK1.1^-^, CD11b^+^, Ly6G^-^, F4/80^-^) counts in the bone marrow, depicted as 10^6^ cells per ml PBS (used to flush femur samples). G: Splenic monocyte (Viab^-^, CD45^+^, CD3^-^, CD19^-^, NK1.1^-^, CD11b^+^, Ly6G^-^, F4/80^-^) numbers per mg of spleen tissue. H-K: Metabolic caging data from mice after HFD feeding H: Respiratory exchange ratio. I: Locomotor activity measured as interruption of infrared (IR) beams. J: Heat dissipation per hour. K: Food intake per 24 hours. A-D; H-K : n=4 per group; E-G: n=5 per group; A-C; E-J: Results are shown as mean ± SEM. D, K: Results are shown as median with IQR. Asterisks indicate statistical significance: * p<0.05; **p<0.01; ns: not statistically significant.

# Supplementary Tables

**Supplementary Table S1:** Sequences of gene specific oligonucleotides used for qPCR analyses

| Gene | forward 5’-3’ | reverse 5’-3’ | reference |
| --- | --- | --- | --- |
| Hydroxymethylbilane synthase (HMBS) | ATGAGGGTGATTCGAGTGGG | TTGTCTCCCGTGGTGGACATA | (1) |
| Glyceraldehyde 3-phosphate dehydrogenase (GAPDH) | CATCATCTCCGCCCCTTCTG | GTGGCAGTGATGGCATGGAC | (2) |
| Acidic ribosomal phosphoprotein P0 (36b4) | GGCCCTGCACTCTCGCTTTC | TGCCAGGACGCGCTTGT | (3) |
| Suppressors of cytokine signaling 3 (SOCS3) | ACCTTCAGCTCCAAAAGCGAGTAC | CGCTCCAGTAGAATCCGCTCTC | (4) |
| Cyclooxygenase-2 (COX2) | ACACACTCTATCACTGGCACC | TTCAGGGAGAAGCGTTTGC | (5) |
| Monocyte chemoattractant protein 1 (MCP-1) | CTCATTCACCAGCAAGATGATCC | CCTTCTTGGGGTCAGCACAG | (6) |
| Regulated And Normal T cell Expressed and Secreted (RANTES) | gctgccctcaccatcatcc | gtattcttgaacccacttcttctctg | (7) |
| Vascular cell adhesion molecule 1 (VCAM-1) | TGAACCCAAACAGAGGCAGAGT | GGTATCCCATCACTTGAGCAGG | (8) |
| Cluster of differentiation 40 (CD40) | TTGTTGACAGCGGTCCATCTA | GCCATCGTGGAGGTACTGTTT | (9) |
| Insulin receptor substrate 1 (IRS1) | CGATGGCTTCTCAGACGTG | CAGCCCGCTTGTTGATGTTG | (9) |
| Insulin receptor substrate 1 (IRS 2) | CTGCGTCCTCTCCCAAAGTG | GGGGTCATGGGCATGTAGC | (9) |
| Glucose transporter type 4 /SLC2A4 (GLUT4) | GTGACTGGAACACTGGTCCTA | CCAGCCACGTTGCATTGTAG | (9) |
| Glucose transporter type 2/SLC2A2 (GLUT2) | TCAGAAGACAAGATCACCGGA | GCTGGTGTGACTGTAAGTGGG | (9) |

# Supplementary References

1. Zhang J, Tang H, Zhang Y, Deng R, Shao L, Liu Y, et al. Identification of suitable reference genes for quantitative RT-PCR during 3T3-L1 adipocyte differentiation. Int J Mol Med. 2014;33(5):1209-18.

2. Lin QQ, Yan CF, Lin R, Zhang JY, Wang WR, Yang LN, et al. SIRT1 regulates TNF-alpha-induced expression of CD40 in 3T3-L1 adipocytes via NF-kappaB pathway. Cytokine. 2012;60(2):447-55.

3. Komiya C, Tsuchiya K, Shiba K, Miyachi Y, Furuke S, Shimazu N, et al. Ipragliflozin Improves Hepatic Steatosis in Obese Mice and Liver Dysfunction in Type 2 Diabetic Patients Irrespective of Body Weight Reduction. PLoS One. 2016;11(3):e0151511.

4. Ding FM, Liao RM, Chen YQ, Xie GG, Zhang PY, Shao P, et al. Upregulation of SOCS3 in lung CD4+ T cells in a mouse model of chronic PA lung infection and suppression of Th17‑mediated neutrophil recruitment in exogenous SOCS3 transfer in vitro. Mol Med Rep. 2017;16(1):778-86.

5. Fornai M, Blandizzi C, Colucci R, Antonioli L, Bernardini N, Segnani C, et al. Role of cyclooxygenases 1 and 2 in the modulation of neuromuscular functions in the distal colon of humans and mice. Gut. 2005;54(5):608-16.

6. Jakkawanpitak C, Hutadilok-Towatana N, Sermwittayawong D. Fungal-like particles and macrophage-conditioned medium are inflammatory elicitors for 3T3-L1 adipocytes. Sci Rep. 2020;10(1):9437.

7. Lean JM, Murphy C, Fuller K, Chambers TJ. CCL9/MIP-1gamma and its receptor CCR1 are the major chemokine ligand/receptor species expressed by osteoclasts. J Cell Biochem. 2002;87(4):386-93.

8. Ren G, Zhao X, Zhang L, Zhang J, L'Huillier A, Ling W, et al. Inflammatory cytokine-induced intercellular adhesion molecule-1 and vascular cell adhesion molecule-1 in mesenchymal stem cells are critical for immunosuppression. J Immunol. 2010;184(5):2321-8.

9. https://pga.mgh.harvard.edu/primerbank/
